# Compressed SENSE in Pediatric Brain Tumor MR Imaging: Assessment of Image Quality, Examination Time and Energy Release

**DOI:** 10.1007/s00062-021-01112-3

**Published:** 2022-01-07

**Authors:** Rieke L. Meister, Michael Groth, Julian H. W. Jürgens, Shuo Zhang, Jan H. Buhk, Jochen Herrmann

**Affiliations:** 1 Department of Diagnostic and Interventional Radiology and Nuclear Medicine, Section of Pediatric Radiology, University Medical Center Hamburg-Eppendorf, Martinistraße 52, 20246 Hamburg, Germany; 2 Philips Healthcare, Hamburg, Germany; 3 Department of Neuroradiology, Asklepios Clinics St. Georg and Wandsbek, Hamburg, Germany

**Keywords:** Acceleration technique, Compressed sensing, Brain neoplasm, Workflow, Specific absorption rate

## Abstract

**Purpose:**

To compare the image quality, examination time, and total energy release of a standardized pediatric brain tumor magnetic resonance imaging (MRI) protocol performed with and without compressed sensitivity encoding (C-SENSE). Recently introduced as an acceleration technique in MRI, we hypothesized that C‑SENSE would improve image quality, reduce the examination time and radiofrequency-induced energy release compared with conventional examination in a pediatric brain tumor protocol.

**Methods:**

This retrospective study included 22 patients aged 2.33–18.83 years with different brain tumor types who had previously undergone conventional MRI examination and underwent follow-up C‑SENSE examination. Both examinations were conducted with a 3.0-Tesla device and included pre-contrast and post-contrast T1-weighted turbo-field-echo, T2-weighted turbo-spin-echo, and fluid-attenuated inversion recovery sequences. Image quality was assessed in four anatomical regions of interest (tumor area, cerebral cortex, basal ganglia, and posterior fossa) using a 5-point scale. Reader preference between the standard and C‑SENSE images was evaluated. The total examination duration and energy deposit were compared based on scanner log file analysis.

**Results:**

Relative to standard examinations, C‑SENSE examinations were characterized by shorter total examination times (26.1 ± 3.93 vs. 22.18 ± 2.31 min; *P* = 0.001), reduced total energy deposit (206.0 ± 19.7 vs. 92.3 ± 18.2 J/kg; *P* < 0.001), and higher image quality (overall *P* < 0.001).

**Conclusion:**

C‑SENSE contributes to the improvement of image quality, reduction of scan times and radiofrequency-induced energy release relative to the standard protocol in pediatric brain tumor MRI.

**Supplementary Information:**

The online version of this article (10.1007/s00062-021-01112-3) contains supplementary material, which is available to authorized users.

## Introduction

Magnetic resonance imaging (MRI) of the brain is a state-of-the-art technique for the visualization of a wide variety of neurological and oncological diseases. Using MRI enables precise anatomical delineation and the differentiation of solid components from cystic areas and necrosis, making it superior to other imaging modalities for the diagnostics and monitoring of treatment response in patients of all ages with central nervous system tumors [1–3]; however, optimal image quality is difficult to ensure in children, who have smaller anatomical structures and show more subtle pathological changes that require the use of high spatial resolution. At the same time, the duration of pediatric imaging examination must be as short as possible to minimize motion artifacts and sedation time [4, 5]. Innovative imaging techniques have been integrated swiftly into pediatric imaging protocols to address these challenges [6, 7].

Recently introduced as an acceleration technology, compressed sensing is poised to gain a foothold in clinical routines [8, 9]. It can be combined with parallel imaging techniques, such as sensitivity encoding (SENSE), and is based on variable density sampling and iterative reconstruction to enable higher spatial resolution and shorter scan duration [6, 10–12]. The balancing of these two aims depends on several factors, including acceleration and regularization factors, as well as coil sensitivity [10, 13, 14]. Several studies have yielded promising results of the combined application of compressed sensing and sensitivity encoding (C-SENSE) in adult populations [8, 9, 15–18], and a few studies have evaluated the application of similar techniques to children, with a primary focus on effects on breathing-dependent scans [6, 19–22].

The purpose of this study was to assess the performance of C‑SENSE as part of a dedicated brain tumor MRI protocol for children. The image quality, examination time, and radiofrequency (RF) energy deposit were assessed.

## Material and Methods

This study was approved by the Institutional Review Board (*Ethikkommission Ärztekammer* Hamburg). Due to the retrospective nature of the study, the requirement for written informed consent was waived.

### Study Cohort

**Fig. 1:**
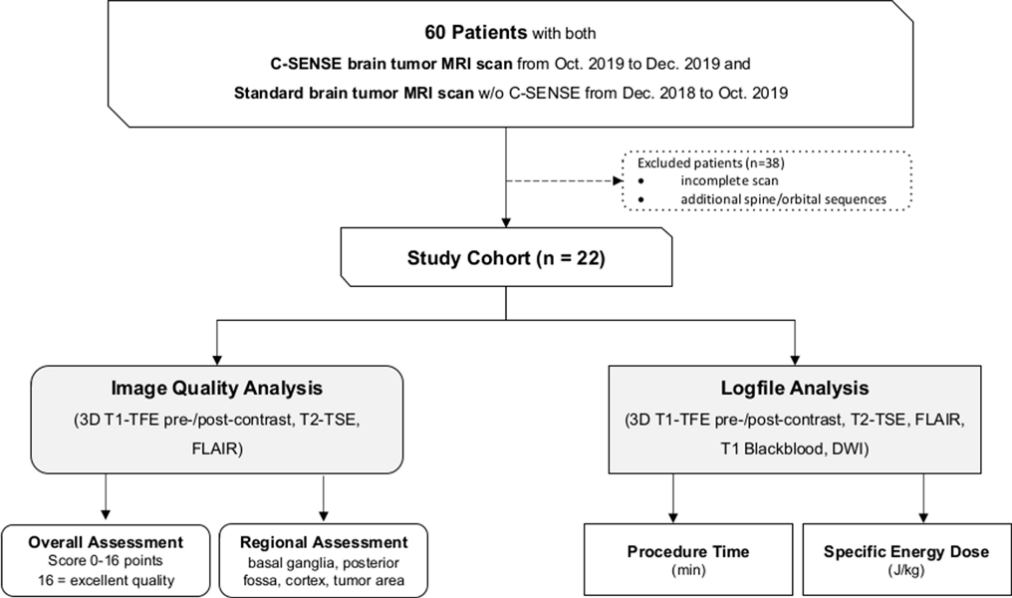
Cohort selection and evaluation pathways. *3D* three-dimensional, *TFE* turbo field echo, *TSE* turbo spin echo, *FLAIR* fluid-attenuated inversion recovery

A total of 60 children with brain tumors who underwent a brain MRI examination with C‑SENSE between October and December 2019 and had undergone at least one previous examination using the standard protocol (without C‑SENSE) were identified retrospectively. Of these patients 28 were excluded due to various criteria in at least 1 of the 2 examinations: variations in protocol or performance of additional imaging sequences, different coil selection, extensive movement with repetition of sequences, incomplete scan. The remaining 22 patients were included in this study. The flow of cohort selection is shown in Fig. 1.

### MRI and Pediatric Brain Tumor Protocol

All MRI examinations were performed on a 3.0-Tesla system (Ingenia, software release R5.6; Philips Healthcare, Best, The Netherlands) with a 32-channel head coil. All patients wore soft ear protection and noise-cancelling headphones during the examination. Foam pads were used to minimize head motion. For unsedated patients, music or video was provided during the examination via the incorporated entertainment system.

Our institution’s basic pediatric brain tumor imaging protocol follows the imaging recommendations of the European Organisation for Research and Treatment of Cancer and the National Brain Tumor Society [23], and includes the following sequences: three-dimensional (3D) T1-weighted turbo-field-echo (T1-TFE), fluid-attenuated inversion recovery (FLAIR), diffusion-weighted imaging (DWI), and T2-weighted turbo-spin-echo (T2-TSE) sequences performed in the axial and coronal planes. The 3D T1-TFE imaging was repeated 3 min after the intravenous injection of gadolinium contrast agent (Dotagraf®, 0.2 ml/kg body weight; Bayer, Leverkusen, Germany).

The corresponding C‑SENSE protocol was performed with the vendor-implemented compressed sensing technology, which employed L1 regularization after wavelet sparsifying transformation and iterative online SENSE reconstruction. It was implemented for all sequences except DWI, due to incompatibility with the echo-planar imaging (EPI) sequence. In addition, the coronal T2-TSE sequence in the standard protocol was replaced with an axial post-contrast black-blood T1-TSE sequence in the C‑SENSE protocol (Supplementary Table 2). All imaging sequences were commercially available and, to maintain consistency of the protocol, the main parameters were kept comparable when possible [24] and optimized when necessary. Details of the protocols are provided in Table 1.

**Table 1:** Parameters of the pediatric brain tumor imaging protocols for standard and C‑SENSE examinations

	3D T1-TFE		T2-TSE		FLAIR	
	Standard	C‑SENSE	Standard	C‑SENSE	Standard	C‑SENSE
FOV (mm^2^)	240 × 240 × 175	240 × 240 × 175	230 × 182 × 152	230 × 182 × 152	230 × 183 × 138	230 × 179 × 152
ACQ Voxel (mm^3^)	1.0 × 1.0 × 1.0	0.85 × 0.85 × 0.85	0.55 × 0.65 × 3.0	0.55 × 0.65 × 3.0	0.65 × 0.87 × 3.0	0.75 × 0.75 × 3.3
REC Voxel (mm^3^)	0.9 × 0.9 × 1.0	0.43 × 0.43 × 0.43	0.4 × 0.4 × 3.0	0.4 × 0.4 × 3.0	0.34 × 0.34 × 3.0	0.34 × 0.34 × 3.3
TR/TE (ms)	8.3/3.8	8.6/4.0	3000/80	3954/80	11,000/125	4800/396
TI (ms)	956.8	989.9	–	–	2800	1650
Acceleration	SENSE 1.2 × 2.2	3.3	–	1.3	SENSE 1.8 × 1.3	4.5
Scan time (min:s)	03:38	03:00	03:36	02:07	03:51	02:38
SNR^a^ (arbitrary)	167.0	145.7	155.3	189.3	205.3	222.3

*C‑SENSE* compressed sensitivity encoding, *3D* three-dimensional, *TFE* turbo field echo, *TSE* turbo spin echo, *FLAIR* fluid-attenuated inversion recovery, *FOV* field of view, *ACQ Voxel * acquisition voxel size, *REC Voxel* reconstruction voxel size, *TR* repetition time, *TE* echo time, *TI* inversion time, *SENSE* sensitivity encoding, *SNR* signal-to-noise ratio

^a^SNR (in arbitrary units) measurements were conducted in a standard phantom with separate noise maps [25] (details see text).

**Table 2:** Image quality ratings

Score	Signal	Contrast	Blurring
0	Non-diagnostic	Flat (very little contrast between parenchymal boarders and CSF)	Blurring of all structures
1	Somewhat limiting	Discrete (little contrast between most structures)	Blurring of most structures
2	Adequate for most structures	Adequate (differentiation of most structures)	Blurring of some structures
3	More than adequate for most structures	Good (sharp for most structures)	Slight blurring
4	More than adequate for most structures	Excellent (sharp for all structures)	No blurring

*CSF* cerebrospinal fluid

### Image Quality

Two board-certified pediatric radiologists (J. H. and M. G., with 16 and 13 years of experience, respectively), who were blinded to the protocol and clinical information, evaluated the images on the hospital’s picture archiving and communication system (PACS; Centricity PACS Universal Viewer, GE web client version 6.0; GE Healthcare, Barrington, IL, USA). Images from individual patients were evaluated with side-by-side comparison of the respective imaging sequences based on consensus reading [6, 26]. Four anatomical areas were chosen for image quality analysis: 1) the infratentorial space, with the posterior fossa, cerebellum, medulla oblongata, and pons; 2) the basal ganglia with surrounding structures and the third ventricle; 3) the cerebral cortex and peripheral supratentorial areas and 4) the tumor region with coverage of its varying appearance. The tissue contrast; visibility and sharpness of major anatomical structures [15, 20, 27], including the border zones of gray and white matter, parenchyma, and cerebrospinal fluid (CSF); and the depiction of small structures, such as the blood vessels, dura, and cranial nerves were rated using a 5-point scale ranging from 0 (non-diagnostic) to 4 (excellent; Table 2). An overall image quality score was calculated for each sequence by summing the four area scores (14–16 = excellent, 11–13 = good, 7–10 = moderate, 0–6 = poor) [28]. The raters also recorded their preference between the standard and C‑SENSE examinations based on their overall subjective impression of usefulness for diagnostic reading (1 = preferred, 0 = not preferred). When neither sequence was preferred, both sequences were given a rating of 0.

The pre-contrast and post-contrast 3D T1-TFE, FLAIR, and axial T2-TSE sequences were included in the image quality analysis. The DWI, coronal T2-TSE, and black-blood T1-TSE sequences were excluded because they were not used in both examinations (Fig. 1). Signal-to-noise-ratio (SNR) characterization was conducted separately with experimental phantom data based on additionally acquired noise maps [25]. Identical parameter settings as in the patient examinations were used for independent quality assurance in both standard and C‑SENSE sequences. The measured SNR values were found comparable between the two protocols (Table 1).

### Total Examination Duration and Energy Deposit

All sequences of both protocols were included in the assessment of total examination duration and energy deposit (Fig. 1). System-generated log files were retrieved and exported with anonymization for offline analysis using software developed in house [9, 29, 30]. The multiple procedural parameters defined for analysis and comparison of the examination duration (Supplementary Fig. 1) included:

- Total table time: overall time that the patient spent on the scanner table.
- Total examination time: the time from the start of the survey scan to the end of the last sequence.
- Total scan time: the overall time spent on active scanning, excluding idle time.
- Total diagnostic scan time: the overall time spent on all diagnostic protocol sequences, excluding survey and calibration scans.
- Total idle time: the overall time spent not planning or scanning, e.g., time spent checking on and communicating with the patient.
- Idle time between scans: the time between scans that was not spent planning or scanning, excluding initial and end idle times.

In addition, the total energy deposit to the patient (in joules/kilogram) during MRI examination was documented as the specific energy dose (SED), defined as the product of the specific absorption rate (SAR) and the sequence acquisition time [9, 29], extracted directly from the log files.

### Statistical Analysis

The data are provided as numbers and percentages for categorical variables and means ± standard deviations (SDs) for continuous variables. The Kolmogorov-Smirnov test, which defines the median SDs and 95% confidence intervals, revealed non-normal distribution of data from the standard and C‑SENSE examinations. The paired Wilcoxon test was performed to compare numeric overall image quality scores, with the null hypothesis that scores would not differ significantly between examinations [6, 19, 20]. The metric variables of total examination duration and energy deposit were compared using the paired *t* test. *P* values < 0.05 were considered to be significant. All statistical analyses were performed with Excel (Microsoft Corporation, Redmond, WA, USA).

## Results

### Study Cohort

Our patient collective consisted of 22 patients (7 females, 15 males; mean age 10.4 ± 4.6 (range 2–19) years), 5 of whom underwent examination under general anesthesia (mean age 4.6 years ± 1.5 months). The main diagnoses leading to MRI examination were astrocytoma (*n* = 8), medulloblastoma (*n* = 4), and ependymoma (*n* = 3), and detailed patient data are provided in Supplementary Table 1. The two consecutive MRI studies were conducted in intervals of 3–6 months following standardized follow-up schemes scheduled by pediatric oncologists.

### Image Quality

**Fig. 2:**
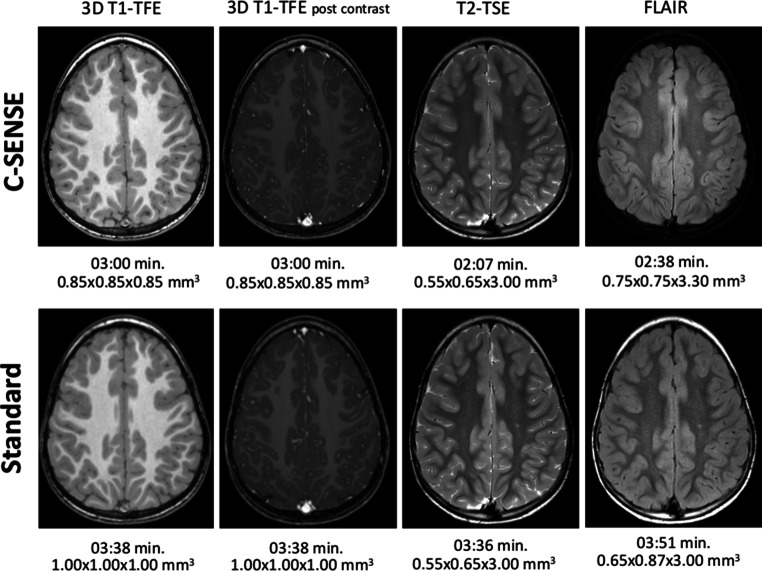
Comparison of images obtained with the standard and C‑SENSE pediatric brain tumor protocols. Example from a 4.8-year-old male patient with ependymoma (*not shown*). Scan times (min) and acquisition voxel sizes (mm^3^) are provided. *3D* three-dimensional, *TFE* turbo field echo, *TSE* turbo spin echo, *FLAIR* fluid-attenuated inversion recovery

In total, 176 datasets were rated in 88 pairs, constituting four sequences of the pediatric brain tumor imaging protocol (pre-contrast and post-contrast 3D T1-TFE, FLAIR, and T2-TSE sequences) of 2 examinations of the 22 patients. Typical examples are presented in Fig. 2. All C‑SENSE and standard examinations had good to excellent image quality (overall scores > 11), with C‑SENSE scores significantly higher than standard examination scores for all sequences (average overall score 13.17 ± 0.79 vs. 11.86 ± 1.11, respectively; *P* < 0.001; Tables 3 and 4).

**Table 3:** Overall image quality and readers’ preference for standard and C‑SENSE pediatric brain tumor MRI examinations

	Image quality			Reader preference
Imaging sequence	Standard	C‑SENSE	*P*	Protocol (*P*)
3D T1-TFE pre-contrast	12.95 ± 0.77	14.27 ± 0.96	< 0.001	C‑SENSE (< 0.001)
T2-TSE	11.68 ± 1.43	12.50 ± 1.53	0.008	C‑SENSE (0.004)
FLAIR	11.36 ± 1.33	12.09 ± 1.24	0.001	C‑SENSE (0.003)
3D T1-TFE post-contrast	11.45 ± 2.93	13.82 ± 1.53	0.001	C‑SENSE (< 0.001)
Average	11.86 ± 1.09	13.17 ± 0.77	< 0.001	C‑SENSE (< 0.001)

Image quality scores: 14–16 = excellent, 11–13 = good, 7–10 = moderate, 0–6 = poor. Scores are given as mean ± SDs; *P* < 0.05 is considered to be significant

*3D* three-dimensional, *TFE* turbo field echo, *TSE* turbo spin echo, *FLAIR* fluid-attenuated inversion recovery

**Table 4:** Regional scores for image quality of standard and C‑SENSE pediatric brain tumor MRI examinations

	Tumor area			Posterior fossa			Cortex			Basal ganglia		
Imaging sequence	Standard	C‑SENSE	*P*	Standard	C‑SENSE	*P*	Standard	C‑SENSE	*P*	Standard	C‑SENSE	*P*
3D T1-TFE pre-contrast	3.14 ± 0.46	3.64 ± 0.48	0.003	2.91 ± 0.29	3.55 ± 0.50	< 0.001	3.86 ± 0.34	4.00 ± 0.00	0.109	3.05 ± 0.55	3.09 ± 0.29	0.285
T2-TSE	2.77 ± 0.67	3.00 ± 0.67	0.063	2.68 ± 0.47	2.82 ± 0.39	0.138	3.73 ± 0.54	3.95 ± 0.21	0.008	2.50 ± 0.47	2.73 ± 0.62	0.091
FLAIR	2.55 ± 0.66	3.00 ± 0.43	0.004	2.50 ± 0.50	2.86 ± 0.46	0.009	3.41 ± 0.58	3.36 ± 0.48	0.423	2.91 ± 0.29	2.86 ± 0.62	0.592
3D T1-TFE post-contrast	3.14 ± 0.95	3.55 ± 0.58	0.063	2.59 ± 0.72	3.32 ± 0.70	< 0.001	3.41 ± 0.89	3.91 ± 0.29	0.007	2.55 ± 0.37	3.05 ± 0.37	< 0.001
Average	2.84 ± 0.74	3.30 ± 0.62	0.003	2.67 ± 0.54	3.14 ± 0.61	< 0.001	3.60 ± 0.65	3.81 ± 0.39	0.006	2.75 ± 0.55	2.93 ± 0.45	0.005

Scores are given as mean ± SD; *P* < 0.05 is considered significant. Image quality scores: 0–4 points with 0 = non-diagnostic and 4 = excellent image quality (see Table 2)

*3D* three-dimensional, *TFE* turbo field echo, *TSE* turbo spin echo, *FLAIR* fluid-attenuated inversion recovery

**Fig. 3:**
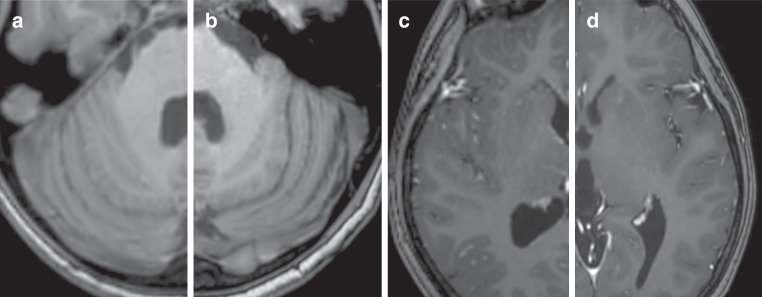
Comparison of 3D T1-TFE images obtained with the standard and C‑SENSE pediatric brain tumor protocols. Example from a 12-year-old male patient with non-germinomatous germ cell tumor (*not shown*). Pre-contrast images of the posterior fossa (**a** standard, **b** C-SENSE) and post-contrast images of the semioval centrum (**c** standard, **d** C-SENSE). The improved spatial resolution of C‑SENSE resulted in better delineation of the arbor vitae cerebelli and cortical vessels with less blurring

**Fig. 4:**
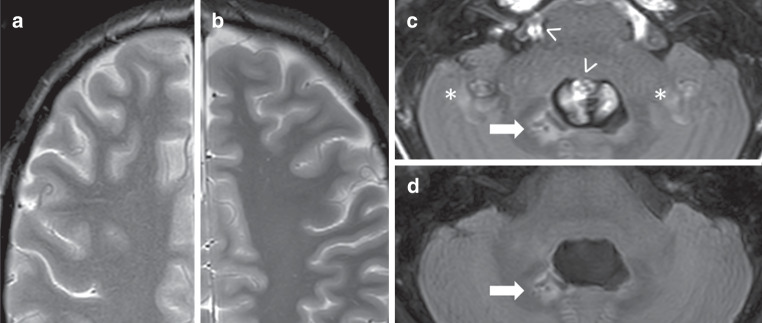
Comparison of T2-TSE and FLAIR images obtained with the standard and C‑SENSE pediatric brain tumor protocols. Example from a 6.4-year-old male patient with infratentorial astrocytoma. Images of the frontal lobes obtained with the T2-TSE sequence in the standard (**a**) and C‑SENSE (**b**) protocols. Note the noisier appearance of the standard examination and similarity of the contrast of gray and white matter and sharpness of smaller structures (e.g., cortical vessels) between sequences. Images of the posterior fossa obtained with the FLAIR sequence in the standard (**c**) and C‑SENSE (**d**) protocols. Due to T1 adjustment, CSF flow artifacts (*arrowhead*) are suppressed sufficiently and their ghosts (*asterisk*) are eliminated in the C‑SENSE image. Pathology is present after astrocytoma resection (*arrow*)

The 3D T1-TFE images were of notably better quality on C‑SENSE than on standard examinations, attributable mainly to increased structure sharpness and higher spatial resolution (Figs. 2 and 3). The C‑SENSE T2-TSE sequences showed better image quality in central brain structures and the same spatial resolution as did standard T2-TSE sequences (Fig. 4). C‑SENSE FLAIR sequences were superior to standard FLAIR sequences in the tumor area and posterior fossa (Table 4), in part because fewer flow artifacts were present (Fig. 4). The readers preferred C‑SENSE images from all sequences in most side-to-side comparisons (64/88 vs. 6/88 pairs; *P* < 0.001). They expressed no protocol preference for 18 pairs of scans.

### Examination Duration and Energy Deposit

All procedural times were significantly shorter for C‑SENSE than for standard examinations (overall *P* < 0.05; Table 5). The greatest differences were found for durations related closely to the scanning technique or imaging sequences, namely the total diagnostic scan time (15.91 ± 1.62 vs. 19.31 ± 1.51 min; *P* < 0.001) and the total scan time (16.52 ± 1.60 vs. 20.94 ± 1.85 min; *P* < 0.001). The energy deposit was significantly lower for C‑SENSE than for standard examinations (SED 92.3 ± 18.2 vs. 206.0 ± 19.7 J/kg; *P* < 0.001).

**Table 5:** Procedural times for standard and C‑SENSE pediatric brain tumor MRI examinations

	Standard	C‑SENSE	Difference (%)	*p*
Table time (min)	29.45 ± 4.66	25.65 ± 2.95	−12.9	0.007
Exam time (min)^a^	26.1 ± 3.93	22.18 ± 2.31	−15.0	0.001
Total scan time (min)^b^	20.94 ± 1.85	16.52 ± 1.60	−21.1	< 0.001
Diagnostic scan time (min)	19.31 ± 1.51	15.91 ± 1.62	−17.6	< 0.001
Total idle time (min)	7.38 ± 3.22	5.45 ± 1.92	−26.2	0.038
Idle time between scans (min)	3.79 ± 2.23	1.86 ± 1.00	−50.8	0.002

*Total table time* overall time patient spents on scanner table, *Total examination time* time from start of survey scan to end of last sequence, *Total scan time* overall time spent on active scanning, *Total diagnostic scan time* overall time spent on all diagnostic protocol sequences, *Total idle time* overall time not spent on planning or scanning, *Idle time between scans* time between scans not spent on planning or scanning

Values given as mean ±1 SD; *P* < 0.05 is considered significant

^a^Excluding initial and end idle times

^b^Sum of all sequence scan times

## Discussion

In this study, we applied C‑SENSE to a dedicated pediatric brain tumor MRI protocol and compared image quality, examination times, and energy deposit to those of standard examinations. The results suggest that C‑SENSE helps to provide superior image quality while reducing procedure times and total energy deposit compared with the conventional method.

As a fundamental part of brain tumor MRI, pre-contrast and post-contrast T1-weighted sequences provide information about the general anatomy and tumor-related blood-brain barrier breakdown via contrast enhancement [23, 24]. A 3D isotropic resolution enables the acquisition of a volumetric dataset and representing all three diagnostically relevant planes with a single scan. The improvement of 3D T1-TFE image quality with C‑SENSE was characterized by the increased sharpness of small structures (i.e., the cerebellum, dura, and intracranial nerves) with no apparent loss of signal or tissue contrast. This property facilitated visual inspection, particularly in contrast-enhancing areas. With the aim of maximizing image quality, implementation of the undersampling and reconstruction algorithm during C‑SENSE 3D T1-TFE sequences reduced the acquisition and reconstruction voxel sizes without loss of the signal-to-noise ratio. This reduction likely contributed to the increased spatial resolution of the sequence.

T2-weighted images help to distinguish between hemorrhage and calcifications, cysts, and solid masses in brain tumor imaging. At the same spatial resolution and with the preservation of contrast, the signal of grey and white matter was slightly more homogeneous on C‑SENSE than on standard T2-TSE images due to the intrinsic denoising capability of C‑SENSE [6, 10–12]. Thus, the readers often preferred the C‑SENSE to the standard T2-TSE images, although image appearance did not differ significantly.

Via CSF suppression, FLAIR images typically aid the detection of vasogenic and cytotoxic edema, gliosis, and gliomatous tumor components. In this study, C‑SENSE had a larger acceleration factor (4.5) than does conventional SENSE (1.8 × 1.3), which led to a slightly noisier image appearance on visual inspection; however, this difference was not deemed impactful for image interpretation. The pseudo-random sampling pattern in the *k* space of C‑SENSE, in combination with the optimized inversion time, helped to reduce flow-related effects (i.e., pulsation artifacts), and readers preferred C‑SENSE over standard FLAIR images.

Relative to standard examinations, C‑SENSE examinations had reduced diagnostic and total scan times (by 17.6% and 21.1%, respectively), attributable directly to the accelerated performance of the four major scan sequences in the imaging protocol. Although not related directly to scan techniques, the shorter total and between-scan idle times in the C‑SENSE examinations could reflect a shorter duration of imaging volume planning, particularly due to the lack of a coronal T2-TSE sequence in the C‑SENSE examination and could be influenced by differences in operator experience. All of these reductions contributed to the significantly shorter procedural duration of the C‑SENSE examinations, as reflected by the reductions in the total examination and table times (by 15.0% and 12.9%, respectively, relative to standard examination).

The significant decrease in the total energy deposit obtained with C‑SENSE relative to standard examination (by 55.2%) in this study can be attributed to reduced sequence acquisition times and thus the lower sequence-specific SED. The SED reduction also could have been affected by the greater undersampling or scan acceleration achieved with C‑SENSE, leading to an assumed decrease of SAR due to less RF excitation and fewer refocusing pulses or shorter echo trains.

Pediatric brain tumor MRI examinations are often challenging because of poor patient cooperation, the need for additional procedures such as sedation, and patients’ smaller anatomical structures. In general, our findings were consistent with previous brain and abdominal imaging studies conducted with adults [9, 15, 16] and abdominal imaging studies conducted with children [6, 19, 20], in which compressed sensing-based technologies were applied to reduce scan times or improve image quality. Regarding the parameter settings of the C‑SENSE protocol, optimization of sequences was conducted during a pilot phase prior to the study, based on our routinely used pediatric brain tumor imaging protocol and existing experience in C‑SENSE applications from literature reports [8, 9, 15–22] as well as other centers. Although this phase was relatively short in order to keep clinical service and patient examinations least disrupted, it represents a typical way for clinical adoption of a new technique in the practice.

The reduction of RF-induced energy in our study is especially advantageous for the examination of sedated or unsedated pediatric patients. To our knowledge, no previous study has examined the amount of RF energy released during pediatric brain tumor MRI examinations. Our findings may help to address concerns about pediatric brain MRI by demonstrating the potential shortening of anesthesia time which could be achieved with C‑SENSE examinations, and which reduces the risks of sedation-related adverse events, airway-related complications, and delayed complications, such as neurotoxicity, particularly in children with severe diseases or disabilities [31–33]. This potential also applies to young patients with brain tumors, who tend to undergo repeated MRI examinations due to the nature of their diseases and surveillance or treatment schemes. The energy deposit reduction may provide a substantial safety benefit for smaller children and newborns, as their limited thermoregulation ability requires careful observation of the RF energy applied during each MRI examination [34, 35]. In addition, shorter examination and procedure times may improve the cooperation of unsedated children and reduce the number of motion-related artifacts [7], as well as enabling the economization of the patient care workflow.

This study has several limitations. First, it was performed at a single institution with a relatively small number of patients, which precluded detailed subgroup analysis according to age, body size, or patient cooperation. In addition, the inclusion of patients with limited types of pathology potentially led to selection bias. Second, image analysis was based on expert consensus and thus did not involve total blinding. As our implementation of C‑SENSE in the pediatric brain tumor protocol was performed with the aim of maximizing clinical utility, the spatial resolution and contrast differed from the standards, and experienced readers could easily identify such differences. Third, the image analysis did not include all images from the brain tumor protocol due to the incompatibility of the C‑SENSE software with EPI-based DWI at the time that this study was conducted. Fourth, the limited sample size and the applied scales were not deemed statistically viable for an interrater analysis, as this method is generally applied in large study cohorts. Consensus reading, however, is considered a solid instrument to clinically assess image quality during protocol amendments for smaller patient collectives. Studies including larger cohorts are desirable to further evaluate the full scope of image quality changes through Compressed SENSE. Fifth, the performance of C‑SENSE examinations months after the standard examinations might have led to the introduction of effects due to patient-related or therapy-related changes. Sixth, differences in operator experience are a factor that was not measured in the current study and is difficult to control in clinical practice.

In conclusion, C‑SENSE implementation in this study not only improved image quality and shortened scan times for pediatric brain tumor MRI, but also contributed to a considerable decrease in energy release, thereby addressing a fundamental concern about pediatric MRI; however, further studies are needed to carefully investigate the clinical impacts of acceleration technologies such as C‑SENSE on energy deposit in children.

## Supplementary Information

*Supplementary Table 1* Patient demographics of the study cohort; *Supplementary Table 2* Pediatric brain tumor MRI protocol* of the study with standard and C-SENSE techniques
