# Supplementary material for: Compressed SENSE in Pediatric Brain Tumor MR Imaging: Assessment of Image Quality, Examination Time and Energy Release
Source: Clin Neuroradiol. 2022 Jan 7;32(3):725–33. doi: 10.1007/s00062-021-01112-3 (PMC9424145; doi:10.1007/s00062-021-01112-3)

**Supplementary Materials**

**Supplementary Table 1.**

**Patient Demographics of the Study Cohort.**

| **Patient** | **Sex** | **Age** | **Histology** | **Months since Diagnosis** |
| --- | --- | --- | --- | --- |
| **P 01** | f | 13.3 | Astrocytoma | 26 |
| **P 02** | m | 8.8 | Medulloblastoma | 25 |
| **P 03** | f | 4.3 | PMNT | 48 |
| **P 04** | f | 12.7 | Astrocytoma | 7 |
| **P 05** | m | 17.8 | Medulloblastoma | 53 |
| **P 06** | m | 8.6 | Astrocytoma | 17 |
| **P 07** | m | 8.9 | Ependymoma | 25 |
| **P 08** | m | 2.3 | Astrocytoma | 10 |
| **P 09** | m | 10.3 | Medulloblastoma | 18 |
| **P 10** | f | 13.3 | Medulloblastoma | 27 |
| **P 11** | m | 5.9 | Astrocytoma | 35 |
| **P 12** | f | 8.6 | Teratoma | 100 |
| **P 13** | m | 9.9 | Astrocytoma | 52 |
| **P 14** | m | 18.1 | Ganglioglioma | 48 |
| **P 15** | m | 18.8 | NGGCT | 9 |
| **P 16** | f | 5.9 | Astrocytoma | 47 |
| **P 17** | m | 8.6 | ATRT | 70 |
| **P 18** | m | 14.1 | Ependymoma | 26 |
| **P 19** | m | 4.8 | Ependymoma | 13 |
| **P 20** | f | 14.2 | Hemangiopericytoma | 5 |
| **P 21** | m | 12.7 | NGGCT | 14 |
| **P 22** | m | 6.4 | Astrocytoma | 57 |

PMNT Peripheral (a)melanotic neuroectodermal tumor of infancy

NGGCT Non-germinomatomous Germ Cell Tumor

ATRT Atypical teratoid rhabdoid tumor

|  | | |
| --- | --- | --- |
| **Supplementary Table 2.**  **Pediatric Brain Tumor MRI Protocol* of the Study with Standard and C-SENSE techniques.** | | |
| **Protocols** | **Standard** | **C-SENSE** |
| Imaging pulse sequences  (sorted according to the scan order) | 3D T1-TFE (pre-contrast) | 3D T1-TFE (pre-contrast) |
|  | DWI | DWI |
|  | T2-TSE coronal | FLAIR |
|  | FLAIR | T2-TSE axial |
|  | T2-TSE axial | *contrast bolus* |
|  | *contrast bolus* | 3D T1-TFE (post-contrast) |
|  | 3D T1-TFE (post-contrast) | T1-TSE black-blood |

* Based on the EORTC-NBTS Recommendations for Glioma Imaging (23).

**Supplementary Figure 1.**

**Procedural times defined and analyzed in the study.**


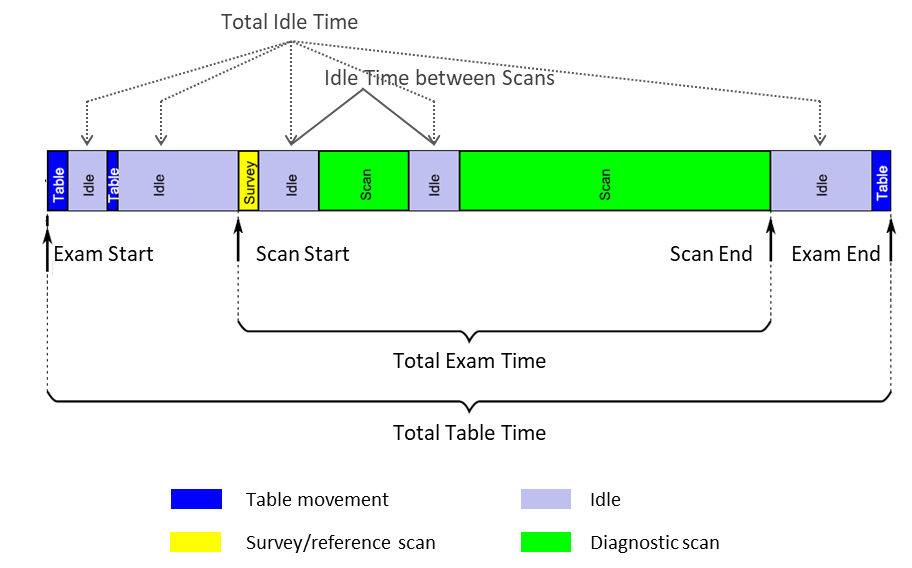

Supplement: Supplementary file 1 — Supplementary Table 1 Patient demographics of the study cohort; Supplementary Table 2 Pediatric brain tumor MRI protocol* of the study with standard and C-SENSE techniques [file 62_2021_1112_MOESM1_ESM.docx]
